# The Role of Genicular Nerve Blocks in Enhancing Postoperative Pain Management After Total Knee Arthroplasty: A Retrospective Study

**DOI:** 10.1155/anrp/8827996

**Published:** 2025-05-06

**Authors:** Yuki Aoyama, Shinichi Sakura, Yoshimi Nakaji, Kornkanok Yuwapattanawong, Tetsuro Nikai

**Affiliations:** ^1^Department of Anesthesiology, Faculty of Medicine, Shimane University, Izumo, Shimane, Japan; ^2^Department of Anesthesiology, Unnan Municipal Hospital, Unnan, Shimane, Japan; ^3^Department of Anesthesiology, Faculty of Medicine, Tokushima University, Tokushima, Tokushima, Japan; ^4^Department of Anesthesiology, Vajira Hospital, Navamindradhiraj University, Bangkok, Thailand

## Abstract

**Background:** Total knee arthroplasty (TKA) is associated with intense postoperative pain, for which continuous femoral triangle block (FTB) and infiltration between the popliteal artery and the capsule of the posterior knee (iPACK) block have been used. Genicular nerves supply sensation to a part of the knee joint that the two blocks do not affect, so we recently started adding genicular nerve blocks (GNBs) to the combination of FTB and iPACK block. In this retrospective study, we examined to see if the addition of GNBs benefited patients undergoing TKA.

**Methods:** We conducted a retrospective analysis of data that had been collected prospectively from patients undergoing TKA and receiving our standard analgesic regimen including continuous FTB and iPACK block in our hospital. We compared patients with and without GNBs regarding intra- and postoperative data including the time needed for block performance, visual analog scale (VAS) pain scores, analgesic requirements, and adverse events. The two-tailed Student's *t* test, Mann–Whitney *U* test, chi-square test, and Fisher's exact test were used for statistical analysis.

**Results:** Seventy-four patients including 41 and 33 patients with and without GNBs, respectively, were evaluated. The demographics of the patients were comparable. VAS pain score at rest on postoperative day 1 was not different between patients who received GNBs and those who did not (20 [0–36] vs. 25 [19–45] in median [IQR], *p* = 0.205). Other measurements related to postoperative pain were also similar throughout the two postoperative days. No severe complications related to blocks were observed.

**Conclusion:** The results of this exploratory retrospective study suggest that the additional benefits of GNBs, if any, are limited for the early postoperative period when combined with continuous FTB and iPACK block in patients undergoing TKA. However, larger, sufficiently powered, and more robust clinical trials are needed to confirm the present results.

## 1. Introduction

Total knee arthroplasty (TKA) is one of the most commonly performed orthopedic procedures. As of 2023, over 80,000 patients underwent the procedure in Japan and the number is expected to increase as our population ages (https://www.joa.or.jp/joa/files/JOANR_annual_report_2022.pdf). However, TKA is associated with significant postoperative pain which disrupts rehabilitation and can be a risk factor for persistent postsurgical pain [1]. Regional anesthesia with less impact on motor function has been shown to offer better postoperative recovery and physical performance and is recommended as a part of multimodal analgesia for TKA [2].

Femoral triangle block (FTB) or adductor canal block and infiltration between the popliteal artery and the capsule of the posterior knee (iPACK) block have recently gained popularity to provide effective analgesia after TKA [3–7]. Adductor canal block and FTB target saphenous nerve with and without the nerve to vastus medialis, respectively, and provide analgesia on the anteromedial aspect of the knee [8, 9]. iPACK block targets the popliteal plexus and other nerve branches from the common peroneal nerve and provides analgesia on the posterior aspect of the knee [5, 10]. These blocks are theoretically motor-sparing and do not disrupt postoperative rehabilitation. Accordingly, we have been using the combination of continuous FTB and iPACK block for TKA. However, these blocks do not cover all the nerves that supply sensation to the knee, especially in some parts of the knee joint, and patients may still experience moderate to severe pain after TKA [5].

Genicular nerves are afferent branches of femoral, sciatic, and obturator nerves and have been described to innervate the anterior knee joint [11–13]. Genicular nerve blocks (GNBs) have been used to help relieve chronic knee pain [14–16] and have recently attracted attention as a possible technique to reduce postoperative pain after TKA [17–19]. Since GNBs target nerve branches innervating the anterior knee joint which cannot be blocked by FTB or adductor canal block and can be performed easily under ultrasound guidance, we recently started adding this technique to the combination of continuous FTB and iPACK block. However, little evidence is available to support our clinical practice of adding GNBs to other peripheral nerve blocks except for one study [18] using a different multimodal analgesic regimen available. Accordingly, in the present retrospective study, we examined to see if the addition of GNBs benefited patients undergoing TKA when combined with our standard analgesic regimen including continuous FTB and iPACK block.

## 2. Patients and Methods

This retrospective cohort study was approved by the Medical Research Ethics Committee, Shimane University Faculty of Medicine on December 14, 2022 (study number 20220207-02), and conducted in accordance with the rules of the Declaration of Helsinki. As a care standard in our department, the intraoperative and postoperative data of patients who received peripheral nerve blocks were prospectively collected and registered in the regional anesthesia database. Registry data include detailed information on block performance, postoperative pain levels, analgesic requirements, motor blockade, and complications in the early postoperative period (for 48 h). We started conducting continuous FTB to replace the femoral nerve block in July 2021 to preserve quadriceps motor function. Thus, we retrieved the data from the registry and the medical records of consecutive patients who underwent unilateral primary TKA between July 2021 and October 2022 under general anesthesia with peripheral nerve blocks including continuous FTB and iPACK block. Written informed consent was waived because the study was limited to the preexisting data. Patients aged more than 18 years with an American Society of Anesthesiologists physical status 1–3 were considered eligible for this study. Exclusion criteria included patients with paralysis or neurological disability on the operating leg, chronic opioid use, allergy to drugs used for our multimodal analgesic regimen, or contraindications to peripheral nerve blocks, received spinal anesthesia and other peripheral nerve blocks, and underwent bilateral and/or revision surgery. Patients were divided into two groups with and without GNBs.

The routine general anesthetic management and block techniques were based on our hospital's care standard. In the operation room, a standard noninvasive monitor was applied and an intravenous line was secured for all patients. Midazolam 1–2 mg and fentanyl 50 μg were intravenously given for sedation before block performance, while the patients remained responsive to verbal cues. The blocks were performed under sterile condition by regional anesthesiologists who were familiar with all the blocks involved in the present study. First, the iPACK block was conducted with a patient in a supine position with the knee joint flexed and the hip externally rotated. A 1–5-MHz convex transducer (LOGIQ e Premium; GE Healthcare, Tokyo, Japan) was placed on the distal thigh to visualize the transverse view of the femoral shaft and medial condyle and the popliteal artery. After the skin was infiltrated with 1% mepivacaine, a 100-mm, 21-gauge block needle (Sonorect needle, CCR; Hakko, Tokyo, Japan) was inserted in-plane under ultrasound guidance, from the anteromedial aspect of the knee toward the space between the popliteal artery and femur. Subsequently, 20 mL of 0.375% ropivacaine was slowly injected while ensuring proper spread of local anesthetic superficial to the posterior capsule. Then, continuous FTB was conducted in-plane approach using a 4–12-MHz linear transducer (LOGIQ e Premium; GE Healthcare, Tokyo, Japan). As previously described [8], the transducer was placed at the mid-thigh level, on the midpoint between the anterior superior iliac spine and the base of the patella, to visualize the short-axis view of the superficial femoral artery underneath the sartorius muscle and saphenous nerve. Following the infiltration of 1% mepivacaine, a catheter-over-needle system with 18-gauge indwelling cannula and 21-gauge Facet tip needle (E-Cath Plus; PAJUNK, Geisingen, Germany) was inserted through the sartorius muscle from lateral to medial direction under ultrasound guidance. Ten milliliters of 0.375% ropivacaine was incrementally injected through the needle to spread around the saphenous nerve and, if seen, the nerve to vastus medialis with intermittent aspiration to avoid intravascular injection. Then, a 20-gauge catheter was inserted through the 18-gauge indwelling cannula in the space made by the local anesthetic. The correct placement of the catheter in the vicinity of the saphenous nerve was verified by injecting a small amount of saline via the catheter. The catheter was fixed with sterile tape (Sorba View SHIELD; Centurion, Williamston, MI).

**Figure 1:**
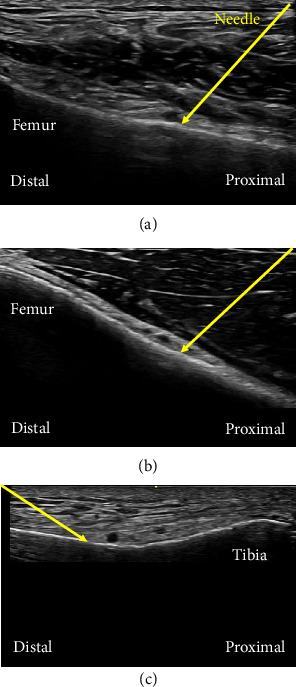
Anatomical and ultrasound images of genicular nerve blocks. Superolateral (a), superomedial (b), and inferomedial (c) genicular nerve blocks were performed. Arrows indicate needle.

GNBs were performed on patients who agreed to receive them. The blocks were conducted on superolateral genicular nerves (SLGN), superomedial genicular nerves (SMGN), and inferomedial genicular nerves (IMGN). Inferolateral GNB was not conducted because of its proximity to the common peroneal nerve and the risk of foot drop [13] that may also occur with a procedure during TKA. A 4–12-MHz linear transducer (LOGIQ e Premium; GE Healthcare, Tokyo, Japan) was placed in a longitudinal orientation over the anterior distal end of the femur or upper tibia to visualize the bone. A 23-gauge needle with a length of 38 mm was advanced under ultrasound guidance until bony contact, adjacent to the genicular arteries if seen (Figures 1(a), 1(b), 1(c)). Three milliliters of 0.375% ropivacaine was injected for each GNB (9 mL in total) to spread along the surface of the femur or tibia at the diaphyseal–metaphyseal transition points.

All patients received general anesthesia after block procedures. General anesthesia was induced and maintained with propofol, fentanyl, remifentanil, and rocuronium. The airway was secured with an endotracheal tube or laryngeal mask. Dexamethasone sodium phosphate 4 mg was intravenously injected at the beginning of surgery, and ondansetron 4 mg and acetaminophen 1 g were given intravenously at the end of surgery. All the surgeries were performed by the same orthopedic team with a tourniquet by a medial parapatellar approach using the navigation system. Intra-articular injection of 0.15% levobupivacaine 80 mL mixed with cefazolin and tranexamic acid was proceeded at skin closure. Continuous infusion of 0.125% levobupivacaine at a rate of 4 mL/h via the FTB catheter was started immediately after surgery with a patient-controlled analgesia (PCA) bolus of 3 mL every 30 min using a disposable ambulatory pump (Rakuraku fuser; Smiths Medical, Tokyo, Japan) and continued for 1–2 days.

After surgery, sugammadex sodium was injected to reverse muscle relaxation, and the endotracheal tube or laryngeal mask was removed when the patient awoke and started breathing adequately. Patients received acetaminophen 1 g every 6 h and loxoprofen sodium hydrate 60 mg every 8 h. Flurbiprofen axetil, diclofenac sodium, and tramadol were used as rescue analgesia when pain was not well controlled despite PCA use. Patients were mobilized and ambulated on postoperative day (POD) 1 and received physical therapy thereafter.

### 2.1. Outcomes

Our hypothesis was that the addition of GNBs could reduce the pain intensity after TKA. The primary endpoint of this study was the pain score at rest in the morning on POD 1. The pain intensity was assessed by visual analog scale (VAS) (0, no pain; 100, worst pain imaginable) at rest and on movement. The secondary endpoints included pain scores on movement, number of PCAs used, requirements for rescue analgesics, side effects, and complications including postoperative nausea and vomiting, motor blockade of the ankle and/or toe, and ambulation with a walker on POD 1. Pain scores and motor blockade were assessed in the early evening (5:00–6:00 p.m.) on POD 0 and the morning (8:00–9:00 a.m.) on PODs 1 and 2 by a member of the regional anesthesia team who did not know whether the patients had received GNBs. The dorsi and plantarflexion of the ankle and toe were assessed by manual muscle testing (MMT; 0, no movement; 5, full range of motion), and the motor block was recorded when MMT was 3 or below. Time required for block performance (time including scanning, preparing block needle to completion of block performance) was also collected.

### 2.2. Statistical Analysis

An a priori sample size calculation was not conducted because this study was conducted retrospectively. Statistical analysis was performed using SPSS 27.0 software for Windows (SPSS Inc., Chicago, IL). The Shapiro–Wilk test was used to check the normality of the data distribution of continuous variables. The two-tailed *t* test was used for parametric statistics, and the values were expressed as mean ± SD. The Mann–Whitney *U* test was applied for nonparametric statistics, and the results were expressed as median (interquartile range). The chi-square test or Fisher's exact test was used for categorical data, and the values were expressed as numbers (percentages). All statistical tests were two-sided, and a *p* value < 0.05 was considered significant. A post hoc power analysis was performed using the *G*^∗^Power software (Version 3.1.9.7; Heinrich Heine University Düsseldorf, Düsseldorf, Germany; https://www.psychologie.hhu.de/arbeitsgruppen/allgemeine-psychologie-und-arbeitspsychologie/gpower).

## 3. Results

**Figure 2:**
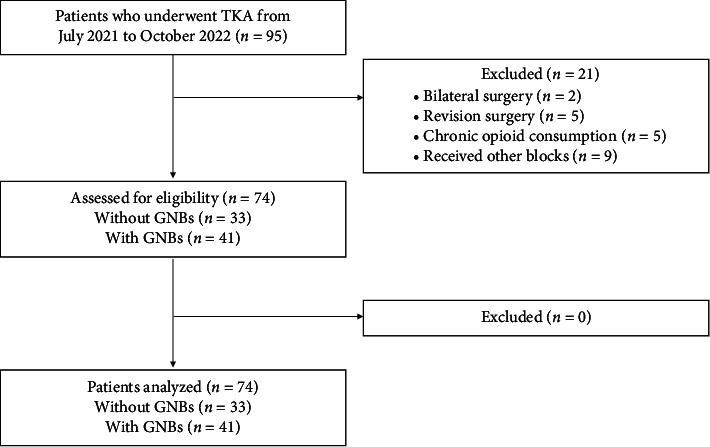
Patient flow diagram. TKA, total knee arthroplasty.

Twenty-one patients were excluded for the reasons shown in Figure 2, and the data of 74 patients (41 and 32 patients with and without GNBs, respectively) were analyzed. The two groups were similar in demographics and surgical characteristics. The opioid consumption during surgery did not differ between the two groups. Additional GNBs took an extra 3 min in median (Table 1).

Patients given GNBs tended to report lower median VAS scores than those who did not receive GNBs on POD 0, but the effect size was small (*r* = 0.060) and no statistical difference was observed (*p*=0.608). The post hoc power analysis revealed that the power was 7.3%. Pain score at rest on POD 1 was not different between patients who received GNBs and those who did not (the post hoc power was 22.9%). VAS pain scores at other time points and the number of PCA bolus injections via the FTB catheter or analgesic requirements were also similar throughout the two PODs (Table 2). Continuous FTB was used for 2 (1–2) and 2 (2) days for patients with and without GNBs, respectively (*p*=0.066). No patient required opioids after surgery. A similar percentage of the patients developed a motor block of the ankle and/or toe on the day of surgery between the two groups (2.4% and 12.1% with and without GNBs, respectively). The percentage of patients ambulating with a walker on POD 1 was also similar. No severe complications related to blocks including local anesthetic toxicity, nerve damage, or fall were observed.

## 4. Discussion

The present retrospective study compared postoperative pain variables after TKA between two groups of patients receiving the combination of continuous FTB and iPACK block with or without GNBs. As opposed to our hypothesis, we found that the pain score on POD 1 was not reduced by adding GNBs. The median VAS pain score on POD 0 was 20 mm lower in patients who received additional GNBs. A difference of 13 mm on the VAS has been estimated to be the minimally clinically important difference [20, 21]; therefore, adding GNBs may have some effect in the early postoperative period. If we were to conduct a randomized comparative study to observe a 13-mm difference in the VAS pain score on POD 0 assuming *α* = 0.05 and *β* = 0.2 (80% power), 113 patients in each group would be required. Postoperative pain scores at other time points, additional analgesic requirement of both PCA and other analgesics, motor weakness in the ankle, and ambulation on POD 1 were similar between patients with or without GNBs.

Regional anesthesia techniques are effective for managing acute pain after TKA. Present guidelines focus on motor-sparling analgesia with good quality of pain relief to facilitate early ambulation [2]. Hence, the trend of regional anesthesia has changed from femoral and sciatic nerve blocks to more distal, motor-sparling blocks such as adductor canal blocks, FTBs, and iPACK blocks [2]. The combination of adductor canal block or FTB and iPACK block can provide reliable analgesia [3–7]; however, patients still experience moderate to severe postoperative pain after TKA [5], suggesting that there remain potential targets for neural blockade.

The anterior aspect of the knee joint is innervated by the nerves to vastus medialis, intermediate, lateralis, and the common and recurrent peroneal nerves, and genicular nerves and the infrapatellar branch. Genicular nerves include SLGN, SMGN, inferolateral genicular nerves (ILGN), and IMGN [11–13]. The effectiveness of GNBs for treating severe chronic pain has been shown in previous studies [14–16]; however, a limited number of studies have evaluated the effect on acute pain after TKA. Akesen et al. [17] conducted a randomized controlled study and showed that GNBs reduced pain scores and morphine consumption in patients after TKA for 24 h. They also reported that GNBs are more effective than iPACK blocks when used the same local anesthetics. Pietrantoni et al. [19] conducted a propensity score–matched study and reported that the analgesic effect of GNBs (using 20 mL of local anesthetic) was noninferior to that of local infiltration analgesia (using 150 mL of the same local anesthetic). These studies would suggest that GNBs are effective analgesia and would be a more suitable technique than single iPACK block and local infiltration analgesia for patients after TKA.

The additional effects of GNBs to other peripheral nerve blocks have previously been evaluated in only one study. Rambhia et al. [18] conducted a randomized-controlled study using sham block and showed that adding GNBs reduced opioid consumption during 48 h and pain score at 6 h. They preoperatively performed 3 GNBs (using 15 mL of 0.25% bupivacaine and dexamethasone 2 mg or sham) and iPACK block and conducted continuous adductor canal block after surgery. They utilized the same combination of peripheral nerve blocks as ours; however, they did not give bolus injection of local anesthetic before starting continuous infusion for adductor canal block. Bolus injection apparently gives more reliable analgesia (because local anesthetic should reach the targeted nerve more easily) and has been used as a standard clinical care. It is possible that the analgesic effect of their adductor canal block was smaller as compared to the FTB used in our study. Besides, other discrepancies in the multimodal analgesia might explain why their results differed from ours. They administered dexamethasone only for the local anesthetic group. Both perineural and intravenous administration of dexamethasone can prolong the duration of peripheral nerve block, reduce pain severity, and decrease opioid consumption [22, 23]. Dexamethasone is now recommended as part of multimodal analgesia after TKA [2]. Therefore, it is likely that the coadministered dexamethasone for the local anesthetic group contributed to better postoperative analgesia in their study. Furthermore, our regimen included an intra-articular local anesthetic injection technique, which has been shown to reduce pain severity and morphine consumption after TKA [24] and, thus, may have masked the effectiveness of GNBs in the present study.

The number of GNBs, dose, and volume of local anesthetic may affect the spread and analgesic effect. We conducted 3 GNBs using 9 mL of local anesthetic, while some previous researchers [16, 19, 25] have conducted more than 4 GNBs using larger volumes. Fonkoue et al. [16] reported that a larger number of injections resulted in better analgesic effect. Therefore, conducting more than 3 GNBs may have produced different results. However, increasing the number of blocks would require a longer time and more needle passes to complete causing additional discomfort for patients.

FTB, iPACK block, and GNB have been thought to be motor-sparling blocks. However, we observed temporal motor blockade of the tibial and/or common peroneal nerve in 2.4% and 12.1% of the patients with and without GNBs, respectively. These results would suggest that, at least, adding 3 GNBs did not increase the risk of motor blockade after TKA. Inferolateral GNB should better be avoided for providing motor-sparling block because it runs close to the common peroneal nerve [13]. iPACK block may not be immune to the motor block. Several cadaveric and clinical studies [10, 26] have reported that motor block can occur after iPACK block because some amount of local anesthetics can possibly spread and affect common peroneal and tibial nerves.

The present study has several limitations. First, this is a retrospective study, which is vulnerable to several types of bias including selection bias and lack of participant blinding. GNBs were performed for patients who agreed to receive them, although the majority of patients consented and received GNBs after we began offering them. Second, we did not identify the location of preoperative and postoperative pain in the knee in each patient. Thus, it is possible that the effect of the addition of GNBs differed depending on the pain location, since GNBs target nerve branches innervating the anterior knee joint. Third, the sample size might be too small to detect the analgesic benefit of GNBs. A larger sample size with more detailed early postoperative data during POD 0 might have been necessary to fully demonstrate the efficacy of adding GNBs to other peripheral nerve blocks. Fourth, the regimens of multimodal analgesia and block techniques involved and the timing of the blocks (i.e., preoperative, intraoperative, and after surgery) might influence the results. Fifth, we conducted catheter insertion for FTB before surgery and did not assess the catheter tip position after surgery. The dislodgement of the catheter tip can occur even though the catheter was properly secured at the skin and could disturb its analgesic effect [27, 28]. Finally, postoperative assessments were conducted within 2 days after surgery. Thus, it is possible, although unlikely, that GNBs generated any difference after the observational period.

## 5. Conclusion

In conclusion, the results of this retrospective study suggest that the additional benefits of GNBs, if any, are limited for the early postoperative period when combined with continuous FTB and iPACK block in patients undergoing TKA. However, the present results were inconclusive due to sample size limitations and needed be confirmed by larger, sufficiently powered, and more robust clinical trials.

## Figures and Tables

**Table 1:** Patient demographics and surgical characteristics.

	Without GNBs (*n* = 33)	With GNBs (*n* = 41)	*p* value	Effect size⁣^∗^
Sex (female), *n* (%)	28 (84.8%)	30 (73.2%)	0.267	0.141
Age (*y*)	74 ± 8	73 ± 8	0.302	0.130
Height (cm)	150.6 ± 7.7	154.1 ± 10.4	0.106	0.382
Body weight (kg)	62.5 ± 12.1	61.1 ± 11.2	0.597	0.124
BMI (kg/m^2^)	27.5 ± 4.6	25.7 ± 4.0	0.070	0.430
ASA-PS (1/2/3) (*n*)	0/30/3	3/36/2	0.316	0.198
Rheumatoid arthritis, *n* (%)	5 (15.2%)	5 (12.2%)	0.744	0.043
Preoperative range of motion				
Knee extension (°)	−10 (−15, −5)	−10 (−15, −5)	0.951	0.007
Knee flexion (°)	110 (95, 120)	120 (110, 125)	0.063	0.216
Surgical site (right), *n* (%)	18 (54.5%)	25 (61.0%)	0.640	0.065
Surgical time (min)	119 (110, 150)	134 (106, 154)	0.625	0.164
Tourniquet time (min)	90 (64, 110)	91 (56, 120)	0.926	0.011
Prosthesis type			0.155	0.224
Posterior stabilized, *n* (%)	24 (72.7%)	22 (53.7%)		
Cruciate retaining, *n* (%)	9 (27.3%)	17 (41.5%)		
Bicruciate preserve, *n* (%)	0 (0%)	2 (4.9%)		
Prosthesis manufacturer			0.652	0.108
Stryker, *n* (%)	31 (93.9%)	38 (92.7%)		
Smith and Nephew, *n* (%)	0 (0%)	1 (2.4%)		
Zimmer, *n* (%)	2 (6.1%)	2 (4.9%)		
Patella replacement, *n* (%)	21 (63.6%)	25 (61.0%)	1.000	0.027
Cemented, *n* (%)	27 (81.8%)	30 (73.2%)	0.419	0.102
Intraoperative fentanyl (μg)	100 (100, 150)	100 (100, 150)	0.613	0.059
Remifentanil (mg)	0.7 (0.5, 1.3)	0.9 (0.6, 1.2)	0.731	0.040
Time for blocks (min)	11 (9, 13)	14 (12, 16)	< 0.0001	0.489

*Note:* Data are presented as number of patients (%), mean ± standard deviation, or median (interquartile range).

Abbreviations: ASA-PS, American Society of Anesthesiologists Physical Status; BMI, body mass index; GNBs, genicular nerve blocks.

⁣^∗^Effect size for continuous variables was reported as Cohen's *d* for parametric variables and *r* for nonparametric variables. For categorical variables, effect size was expressed using Phi and Cramér's *V*.

**Table 2:** Postoperative patient data regarding pain scores, postoperative analgesic requirements, and adverse events.

	Without GNBs (*n* = 33)	With GNBs (*n* = 41)	*p* value	Effect size⁣^∗^
VAS at rest (mm)				
POD 0	40 (0, 60)	20 (0, 70)	0.608	0.060
POD 1	25 (19, 45)	20 (0, 36)	0.205	0.147
POD 2	30 (13, 49)	20 (0, 30)	0.212	0.145
VAS on movement (mm)				
POD 1	60 (40, 80)	40 (25, 79)	0.194	0.151
POD 2	55 (40, 60)	50 (38, 73)	0.884	0.017
PCA via the catheter (time)				
POD 1	1 (0, 3)	0 (0, 3)	0.573	0.066
POD 2	0 (0)	0 (0, 3)	0.062	0.217
Rescue analgesics required *n* (%)				
POD 0	9 (27.3%)	5 (12.2%)	0.137	0.191
POD 1	10 (30.3%)	9 (22.0%)	0.436	0.095
POD 2	2 (6.1%)	4 (9.8%)	0.686	0.067
Presence of PONV, *n* (%)	6 (18.2%)	6 (14.6%)	0.757	0.048
Presence of motor blockade, *n* (%)	4 (12.1%)	1 (2.4%)	0.165	0.192
Ambulation on POD 1, *n* (%)	16 (48.5%)	20 (48.8%)	1.000	0.003

*Note:* Data are presented as median (interquartile range) or number of patients (%). POD, postoperative day; PONV, postoperative nausea and vomiting.

Abbreviations: PCA, patient-controlled analgesia; VAS, visual analog scale.

⁣^∗^Effect size was reported as *r* for continuous variables and Phi for categorical variables.

## Data Availability

The data that support the findings of this study are available on request from the corresponding author. The data are not publicly available due to privacy or ethical restrictions.

## Nomenclature

TKA: Total knee arthroplasty
FTB: Femoral triangle block
iPACK: Infiltration between the popliteal artery and the capsule of the posterior knee
PCA: Patient-controlled analgesia
POD: Postoperative day
VAS: Visual analog scale
MMT: Manual muscle testing
SLGN: Superolateral genicular nerves
SMGN: Superomedial genicular nerves
ILGN: Inferolateral genicular nerves
IMGN: Inferomedial genicular nerves
BMI: Body mass index
ASA-PS: American Society of Anesthesiologists Physical Status
PONV: Postoperative nausea and vomiting

## References

[1] Grosu I., Lavand’homme P., Thienpont E. (2014). Pain After Knee Arthroplasty: an Unresolved Issue. *Knee Surgery, Sports Traumatology, Arthroscopy: Official Journal of the ESSKA*.

[2] Lavand’homme P. M., Kehlet H., Rawal N., Joshi G. P. (2022). Pain Management After Total Knee Arthroplasty: PROcedure SPEcific Postoperative Pain ManagemenT Recommendations. *European Journal of Anaesthesiology*.

[3] Thobhani S., Scalercio L., Elliott C. E. (2017). Novel Regional Techniques for Total Knee Arthroplasty Promote Reduced Hospital Length of Stay: An Analysis of 106 Patients. *The Ochsner Journal*.

[4] Kim D. H., Beathe J. C., Lin Y. (2019). Addition of Infiltration Between the Popliteal Artery and the Capsule of the Posterior Knee and Adductor Canal Block to Periarticular Injection Enhances Postoperative Pain Control in Total Knee Arthroplasty: A Randomized Controlled Trial. *Anesthesia & Analgesia*.

[5] Ochroch J., Qi V., Badiola I. (2020). Analgesic Efficacy of Adding the IPACK Block to a Multimodal Analgesia Protocol for Primary Total Knee Arthroplasty. *Regional Anesthesia and Pain Medicine*.

[6] Tang X., Jiang X., Lei L. (2022). IPACK (Interspace Between the Popliteal Artery and the Capsule of the Posterior Knee) Block Combined With SACB (Single Adductor Canal Block) Versus SACB for Analgesia After Total Knee Arthroplasty. *Orthopaedic Surgery*.

[7] Hussain N., Brull R., Sheehy B., Dasu M., Weaver T., Abdallah F. W. (2021). Does the Addition of iPACK to Adductor Canal Block in the Presence or Absence of Periarticular Local Anesthetic Infiltration Improve Analgesic and Functional Outcomes Following Total Knee Arthroplasty? A Systematic Review and Meta-Analysis. *Regional Anesthesia and Pain Medicine*.

[8] Bendtsen T. F., Moriggl B., Chan V., Børglum J. (2016). The Optimal Analgesic Block for Total Knee Arthroplasty. *Regional Anesthesia and Pain Medicine*.

[9] Woodworth G. E., Arner A., Nelsen S., Nada E., Elkassabany N. M. (2023). Pro and Con: How Important is the Exact Location of Adductor Canal and Femoral Triangle Blocks?. *Anesthesia & Analgesia*.

[10] Niesen A. D., Harris D. J., Johnson C. S. (2019). Interspace Between Popliteal Artery and Posterior Capsule of the Knee (IPACK) Injectate Spread: A Cadaver Study. *Journal of Ultrasound in Medicine*.

[11] Tran J., Peng P. W. H., Lam K., Baig E., Agur A. M. R., Gofeld M. (2018). Anatomical Study of the Innervation of Anterior Knee Joint Capsule: Implication for Image-Guided Intervention. *Regional Anesthesia and Pain Medicine*.

[12] Fonkoué L., Behets C., Kouassi J. É. K. (2019). Distribution of Sensory Nerves Supplying the Knee Joint Capsule and Implications for Genicular Blockade and Radiofrequency Ablation: An Anatomical Study. *Surgical and Radiologic Anatomy*.

[13] McCormick Z. L., Cohen S. P., Walega D. R., Kohan L. (2021). Technical Considerations for Genicular Nerve Radiofrequency Ablation: Optimizing Outcomes. *Regional Anesthesia and Pain Medicine*.

[14] Elsaman A. M., Maaty A., Hamed A. (2021). Genicular Nerve Block in Rheumatoid Arthritis: A Randomized Clinical Trial. *Clinical Rheumatology*.

[15] Shanahan E. M., Robinson L., Lyne S. (2023). Genicular Nerve Block for Pain Management in Patients with Knee Osteoarthritis:A Randomized Placebo-Controlled Trial. *Arthritis & Rheumatology*.

[16] Fonkoue L., Steyaert A., Kouame J. E. K. (2021). A Comparison of Genicular Nerve Blockade With Corticosteroids Using Either Classical Anatomical Targets Vs Revised Targets for Pain and Function in Knee Osteoarthritis: A Double-Blind, Randomized Controlled Trial. *Pain Medicine*.

[17] Akesen S., Akesen B., Atıcı T., Gurbet A., Ermutlu C., Özyalçın A. (2021). Comparison of Efficacy Between the Genicular Nerve Block and the Popliteal Artery and the Capsule of the Posterior Knee (IPACK) Block for Total Knee Replacement Surgery: A Prospective Randomized Controlled Study. *Acta Orthopaedica et Traumatologica Turcica*.

[18] Rambhia M., Chen A., Kumar A. H., Bullock W. M., Bolognesi M., Gadsden J. (2021). Ultrasound-Guided Genicular Nerve Blocks Following Total Knee Arthroplasty: A Randomized, Double-Blind, Placebo-Controlled Trial. *Regional Anesthesia and Pain Medicine*.

[19] Pietrantoni P., Cuñat T., Nuevo-Gayoso M. (2021). Ultrasound-guided Genicular Nerves Block: An Analgesic Alternative to Local Infiltration Analgesia for Total Knee Arthroplasty: A Noninferiority, Matched Cohort Study. *European Journal of Anaesthesiology*.

[20] Gallagher E. J., Liebman M., Bijur P. E. (2001). Prospective Validation of Clinically Important Changes in Pain Severity Measured on a Visual Analog Scale. *Annals of Emergency Medicine*.

[21] Cepeda S. M., Africano J. M., Polo R., Alcala R., Carr D. B. (2003). What Decline in Pain Intensity is Meaningful to Patients With Acute Pain?. *Pain*.

[22] Waldron N. H., Jones C. A., Gan T. J., Allen T. K., Habib A. S. (2013). Impact of Perioperative Dexamethasone on Postoperative Analgesia and Side-Effects: Systematic Review and Meta-Analysis. *British Journal of Anaesthesia*.

[23] Heesen M., Klimek M., Imberger G., Hoeks S. E., Rossaint R., Straube S. (2018). Co-Administration of Dexamethasone With Peripheral Nerve Block: Intravenous Vs Perineural Application: Systematic Review, Meta-Analysis, Meta-Regression and Trial-Sequential Analysis. *British Journal of Anaesthesia*.

[24] Karlsen A. P. H., Wetterslev M., Hansen S. E., Hansen M. S., Mathiesen O., Dahl J. B. (2017). Postoperative Pain Treatment After Total Knee Arthroplasty: A Systematic Review. *PLoS One*.

[25] Caldwell G. L., Selepec M. A. (2019). Reduced Opioid Use After Surgeon-Administered Genicular Nerve Block for Anterior Cruciate Ligament Reconstruction in Adults and Adolescents. *The Musculoskeletal Journal of Hospital for Special Surgery*.

[26] Kampitak W., Tanavalee A., Ngarmukos S., Tantavisut S. (2020). Motor-Sparing Effect of iPACK (Interspace Between the Popliteal Artery and Capsule of the Posterior Knee) Block Versus Tibial Nerve Block After Total Knee Arthroplasty: A Randomized Controlled Trial. *Regional Anesthesia and Pain Medicine*.

[27] Aoyama Y., Sakura S., Kitajo A., Saito Y. (2021). Incidence and Effects of Postoperative Migration of Interscalene Catheter Tips Placed Using Ultrasound-Guided Anterior and Posterior Approaches. *Journal of Anesthesia*.

[28] Fujino T., Yoshida T., Kawagoe I., Hinotsume A., Hiratsuka T., Nakamoto T. (2023). Migration Rate of Proximal Adductor Canal Block Catheters Placed Parallel Versus Perpendicular to the Nerve After Total Knee Arthroplasty: A Randomized Controlled Study. *Regional Anesthesia and Pain Medicine*.

